# Acute Gastrointestinal Injury in Polytrauma: Special Attention to Elderly Patients

**DOI:** 10.7150/ijms.98997

**Published:** 2024-08-26

**Authors:** Cong Zhang, Teding Chang, Deng Chen, Jialiu Luo, Shunyao Chen, Peidong Zhang, Zhiqiang Lin, Jian Luo, Quan Zhou, Wenguo Wang, Huaqiang Xu, Liming Dong, Zhaohui Tang

**Affiliations:** 1 Department of Trauma Surgery, Emergency Surgery & Surgical Critical, Tongji Trauma Center, Tongji Hospital, Tongji Medical College, Huazhong University of Science and Technology, Wuhan 430030, China.; 2 Department of Emergency and Critical Care Medicine, Tongji Hospital, Tongji Medical College, Huazhong University of Science and Technology, Wuhan 430030, China.; 3 Department of Geriatrics, Tongji Hospital, Tongji Medical College, Huazhong University of Science and Technology, Wuhan 430030, China.; 4 Intensive Care Unit, Trauma Center, Suizhou Central Hospital, Hubei University of Medicine, Suizhou 441300, China.

**Keywords:** Polytrauma, Elderly Patients, Acute Gastrointestinal Injury, Retrospective Study

## Abstract

**Background:** Acute gastrointestinal injury (AGI) has been documented in critically ill patients, yet there remains a dearth of knowledge regarding its occurrence, predisposing factors, and outcomes in elderly polytrauma patients, a significant but overlooked population. This study aims to examine the frequency, risk factors, and clinical implications of AGI in elderly polytrauma patients.

**Methods:** A retrospective, observational, multicenter study was carried out in two Level I trauma centers, encompassing a cohort of 1054 polytrauma patients from July 2020 to April 2022.

**Results:** A total of 965 consecutive polytrauma patients were recruited who were divided into youth group (n=746) and elderly group (n=219). 73.5% of elderly patients after polytrauma were accompanied by AGI. An increasing ISS (OR=2.957, 95%CI: 1.285-7.714), SI (OR=2.861, 95%CI: 1.372-5.823), serum lactate (OR=2.547, 95%CI: 1.254-5.028), IL-6 (OR=1.771, 95%CI: 1.145-8.768), APTT (OR=1.462, 95%CI: 1.364-4.254) and a decreasing GCS (OR=0.325, 95%CI: 0.116-0.906) were each associated with an increasing risk of AGI in elderly polytrauma patients. Elderly polytrauma patients with AGI were presented relatively high 28-day mortality (40.4%) and super high 60-day mortality (61.2%) compared with elderly group without AGI and youth group with AGI. The area under the curve for predicting 28-day mortality in elderly polytrauma patients with AGI was 0.93 for AGI-III,IV with 96% sensitivity and 87% specificity.

**Conclusion:** Elderly patients have a higher incidence and a worse prognosis of AGI after polytrauma. ISS, GCS, SI, serum lactate, IL-6, and APTT are identified as reliable prognostic markers to distinguish the AGI and N-AGI in elderly polytrauma patients. AGI-III,IV was the independent predictor of mortality in elderly polytrauma patients with AGI.

## 1. Introduction

Polytrauma, characterized by adverse outcomes and elevated mortality rates resulting from severe injuries and persistent complications, remains a multifaceted health concern, particularly among the aging population^1^. The aging demographic landscape in Chinese society is leading to an anticipated rise in the number of elderly individuals requiring trauma care. Moreover, the increasing engagement in vigorous activities by elderly patients is contributing to a higher incidence of severe injuries within this demographic, a trend that is projected to persist in the coming years^2^. The emergence of these advancements presents a formidable obstacle for trauma care providers globally, as advanced biological age has been recognized as a significant individual risk factor for adverse outcomes in trauma cases. Delivering trauma care to elderly individuals is notably intricate and demanding due to their frequent presentation in a fragile state, rendering them more susceptible to complications irrespective of the severity of their injuries^3^.

Despite reduced frequency of the multiple organ dysfunction syndrome (MODS) after polytrauma has been reported in the last decades, it still remains a major cause of death after severe trauma. MODS has a higher frequency in elderly patients than their younger counterparts due to the decline of physiological function and preinjury comorbidities 4. The gut has been hypothesized to be the motor of MODS and the critical role of gastrointestinal failure plays in MODS has been recognized for years^5^. Under multiple insults such as trauma, infection and shock, the gastrointestinal tract is not only the "target" organ that is damaged, but also the "trigger" organ that aggravates the injury^6^. A recent multicenter study found that acute gastrointestinal injury (AGI) is a common occurrence in critically ill patients, with the severity of AGI being positively correlated with 28- and 60-day mortality rates^7^. Furthermore, up to 80% of elderly patients may exhibit various signs of AGI, which is linked to increased mortality^7^. However, the pathophysiological significance of gastrointestinal dysfunction in the progression of multiple organ dysfunction syndrome (MODS) has not been thoroughly explored.

Trauma surgeons pay more attention to cardiac, renal or respiratory dysfunction, and even coagulation disorders in elderly polytrauma patients^8^. By contrast, AGI is rarely included in the everyday vocabulary of trauma specialists. A major limitation of assessment of AGI is the lack of markers for evaluating gastrointestinal function, and some of the symptoms are subjective and poorly defined^7^. So, the assessment of gastrointestinal injury is often ignored. Limited clinical data exists on the occurrence of acute gastrointestinal injury (AGI) following polytrauma, particularly in elderly patients. To address this gap in knowledge, a multicenter retrospective cohort study was conducted to determine the incidence of AGI, identify risk factors, and assess its impact on in-hospital mortality in elderly polytrauma patients. The primary objective of this study was to test the hypothesis that AGI has a higher incidence in elderly polytrauma patients and worsens the prognosis, ultimately, to attract special attention on AGI in elderly polytrauma patients.

## 2. Materials and Methods

### 2.1. Study design and Patients

This study was a retrospective, multi-center, observational trial approved by the institutional review board, with informed consent obtained from all participants. The study included patients admitted to the traumatic intensive care unit (TICU) or intensive care unit (ICU) at Tongji Hospital and Suizhou Central Hospital between July 2020 and April 2022.

The inclusion criteria were as follows: (1) age >18 years; (2) meet the new “Berlin definition” of polytrauma: injuries with an Abbreviated Injury Scale (AIS) score of 3 or higher in at least two body regions (2AIS ≥ 3)^9^; 3) admission within 24 hour; 4) including laboratory values (IL-6,PCT,CRP and serum lactate) within 48 hour. The exclusion criteria were: 1) stomach, intestinal medical or primary injury to the gastrointestinal tract at admission; 2) terminal malignant tumor; 3) hormones or immune preparations used; 4) missing clinical records. A total of 1054 enrolled patients were diagnosed with polytrauma within 24 hours after injury. Among them, 89 patients were excluded due to lack of complete information or an unclear AGI classification. All enrolled patients were divided into 2 groups according to their age, the elderly group (age≥65 years) and the youth group (18<age<65 years). Moreover, patients after polytrauma were divided into the AGI group and the N-AGI group based on the occurrence of AGI or not.

### 2.2. Ethical statement

This research was granted approval by the Institutional Review Board of Tongji Hospital at Huazhong University of Science and Technology (IRB number: TJ-IRB20200720; approval date: 22 July 2020) and the Institutional Review Board of Suizhou Central Hospital (IRB number: none; approval date: 1 June 2019). The study adhered to the principles outlined in the Declaration of Helsinki, and informed consent was obtained from all participants or their legally authorized representatives.

### 2.3. Diagnosis

The diagnosis of acute gastrointestinal injury (AGI) was determined based on the definition proposed by the Working Group on Abdominal Problems (WGAP) of the European Society of Intensive Care Medicine (ESICM) in 2012^10^. The daily assessment of AGI grade during hospitalization followed the recommendations of the ESICM grading system (Table S1). Complications were diagnosed during hospitalization and their diagnosis was based on established definitions^11^. The clinical diagnosis standard for persistent inflammation immunosuppression catabolism syndrome (PICS) were as follows: (1) Admission to the ICU > 14 days; (2) CRP >50 mg/L; (3) Total lymphocyte count < 0.80 ×109/L; (4) Serum albumin < 30 g/L, Pre-albumin <100 mg/L, Creatinine height index < 80%, Weight loss > 10% 'or' BMI < 18 during hospitalization^12^.

### 2.4. Data Collection

The baseline clinical characteristics of patients within the first 48 hours of admission were retrospectively collected from electronic medical and nursing records. These characteristics included age, sex, mechanisms of injury, injury regions, Glasgow Coma Scale (GCS), Injury Severity Score (ISS), Shock Index (SI), laboratory values (IL-6, PCT, APTT, serum lactate, etc.), and discharge records. Additionally, events during the hospital course such as acute gastrointestinal injury (AGI), acute respiratory distress syndrome (ARDS), acute kidney injury (AKI), hospital-acquired infections (HAI), multiple organ failure (MOF), sepsis, length of ICU stay, duration of ventilator use and death were documented^10^ ^11^.

Regular post-discharge appointments were arranged at 1 month and 2 months intervals, with additional visits scheduled as needed. In cases where a patient passed away during the 2-month follow-up period, the reasons for their death were ascertained through communication with their designated representatives and confirmed by death certificates acquired from the relevant local public health authorities. Patients for whom this information could not be obtained were categorized as "lost to follow-up".

### 2.5. Study End Points

The primary outcome of this study was to determine the incidence and risk factors of AGI in elderly patients after polytrauma. Secondary outcomes were to determine the complications and mortality in elderly polytrauma patients with AGI.

### 2.6. Statistical Analysis

Before conducting the analysis, the data underwent scrutiny for normality and homogeneity of variance. Categorical variables were assessed using frequency counts and percentages, while continuous variables were evaluated using either median and interquartile range (IQR) or mean plus standard deviation (mean ± SD). Statistical tests such as the Student t-test, Mann-Whitney U test, and χ2 were employed to compare continuous and categorical variables. Furthermore, a multivariable logistic regression analysis was conducted to ascertain the risk factors associated with mortality within 28 days and acute gastrointestinal injury (AGI) in elderly polytrauma patients. An ROC curve was constructed to assess the prognostic value for elderly trauma patients. Statistical significance was determined at p<0.05. Data analysis was conducted using SPSS 23.0 and visualized using GraphPad Prism software 9.3.1.

## 3. Results

### 3.1 Characteristics of Polytrauma Patients

**Figure 1:**
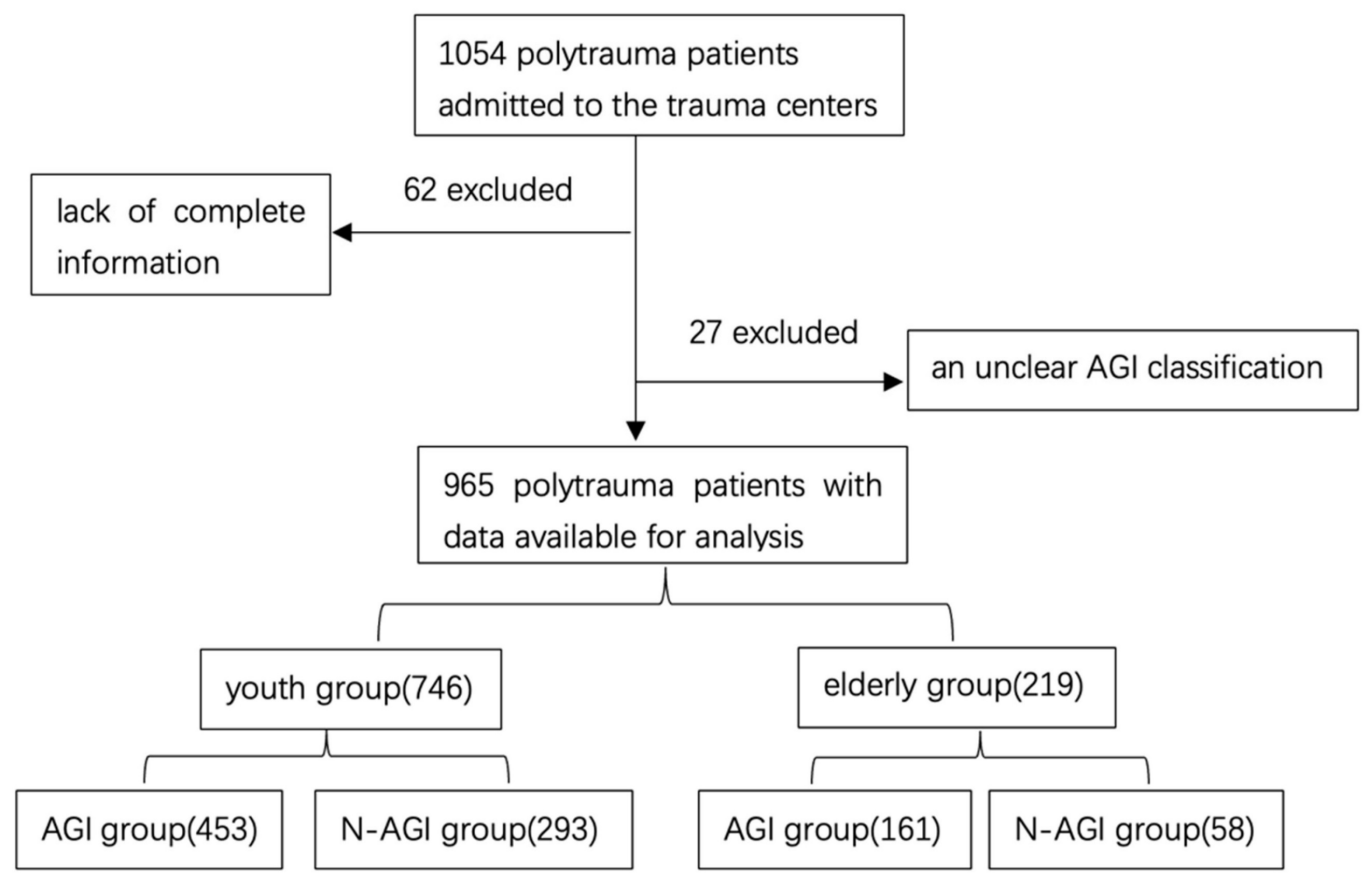
Case identification procedure.

Between July 2020 and April 2022, a cohort of 1054 polytrauma patients were admitted to trauma centers. Following exclusion criteria, 965 consecutive polytrauma patients were included in the study, with 746 categorized in the youth group and 219 in the elderly group (Figure 1). Demographics and characteristics of these patients are detailed in Table 1.

A total of 965 consecutive polytrauma patients were recruited from the two Level I trauma centers (66.7% male; mean age: 50.0±7.6). The mean value of Injury Severity Score (ISS) was 30.9, while for Glasgow coma scale (GCS) and Shock index (SI) were 10.4 and 0.9 respectively, indicating a severely injured population. Overall, 55.6% (537/965) patients suffered head injury, 40.4% (390/965) patients had a thorax injury, 33.4% (319/965) suffered spine injury, and 21.2% (205/965) suffered pelvis injury, while lower-extremity and upper-extremity were recorded for 58.4% (564/964) and 45.1% (435/965) patients, respectively. The injury was mainly caused by traffic accident 70.7% (682/965), followed by high-energy fall (22.7%, 219/965), and other types of accidents (6.6%, 64/965). There were no differences between the elderly group and youth group for Gender (P=0.955), ISS (P=0.062), GCS(P=0.084), SI (P=0.056), Cause of injury (P=0.125), and Injury site (P =0.523) (Table 1).

### 3.2 Incidence of AGI in Patients after polytrauma

The overall incidence of AGI among all enrolled patients after polytrauma was 63.6% (614/965). The incidence of AGI in elderly patients after polytrauma was 73.5% (161/219), among which the distribution of the AGI grades was 14.9% for grade I (n = 24), 39.8% for grade II (n = 64), 35.4% for grade III (n = 57), and 9.9% for grade IV (n=16). There were significant differences in incidence of AGI between the youth group and elderly group. The elderly polytrauma patients had higher incidence of AGI (73.5% vs.60.7%, P<0.001) than youth individuals. As for the distribution of the AGI grades, the elderly group had higher ratio of severe AGI (AGIIII, IV) (45.3% vs. 11.4%, P<0.001) than youth group (Table 1).

### 3.3 Early Risk Factors for AGI in elderly polytrauma patients

Table 2 shows the comparison between elderly polytrauma patients with and without AGI in univariate analysis. When compared with patients without AGI, those with AGI had a higher ISS, higher SI, higher levels of heart rate, serum lactate, PCT, IL-6 and APTT, higher ratio of ABE≤-6 and INR≥1.4, and lower GCS (P<0.05), which were retrospectively collected in the first 48 hours after admission. There were no differences in age (P = 0.101), gender (P = 0.646) between the AGI group and N-AGI group (Table 2). The incidence of PICS in elderly patients after polytrauma was 18.3% (n=40). The AGI group had higher incidence of PICS (24.2% vs.1.7%, P<0.001) than N-AGI group. In addition, the duration of ICU and mechanical ventilation were significantly (P<0.001) different between the AGI group and N-AGI group. The elderly patients after polytrauma with AGI had higher ratio of mechanical ventilation (70.2% vs. 29.3%, P<0.001), use of vasoactive drug (60.9% vs. 24.1%, P<0.001) and longer of ICU stays (15 vs. 6, P<0.001) (Table 2).

Following univariate analysis, variables with a P value <0.05 were chosen for inclusion in a multivariate analysis utilizing a multiple logistic regression model. The results of the multivariate logistic regression analysis indicated that an elevated Injury Severity Score (ISS) (OR = 2.957, 95% CI: 1.285-7.714), Shock Index (SI) (OR = 2.861, 95% CI: 1.372-5.823), serum lactate level (OR = 2.547, 95% CI: 1.254-5.028), interleukin-6 (IL-6) levels (OR = 1.771, 95% CI: 1.145-8.768), activated partial thromboplastin time (APTT) (OR = 1.462, 95% CI: 1.364-4.254), and a decreased Glasgow Coma Scale (GCS) score (OR = 0.325, 95% CI: 0.116-0.906) were all independently associated with an increased risk of acute gastrointestinal injury (AGI) in elderly polytrauma patients (Table 3).

### 3.4 Complications and mortality in elderly polytrauma patients with AGI

Regarding of mortality, various trends were observed among the different groups which the presence of one or both of advanced age and AGI increased the mortality, as shown in Table 4. For patients in the elderly group with AGI and without AGI, elderly group with AGI were presented relatively higher 28-day mortality (40.4% vs. 24.1%) and higher 60-day mortality (61.2% vs. 36.2%) than elderly group without AGI. When compared with youth group with AGI, elderly group with AGI were presented relatively higher 28-day mortality (40.4% vs. 9.1%) and higher 60-day mortality (61.2% vs. 15.7%), (p<0.001, Table 4). In addition, various trends were observed among the different groups concerning hospitalization complications. The incidence of hospitalization complications in elderly group with AGI was significantly higher than that in elderly group without AGI and youth group with AGI, significantly (p<0.001). The four most frequent hospitalization complications in elderly group with AGI were hospital acquired infections (HAI, 88.2%), multi-organ failure (MOF, 66.5%), respiratory distress syndrome (ARDS, 56.5%) and sepsis (41.6%).

### 3.5 Risk Factors for death within 28 days in elderly polytrauma patients with AGI

Risk factors for 28-days death in elderly polytrauma patients with AGI have been thoroughly analyzed. Univariable analysis of risk factors is shown in Table 5. The Age, ISS, GCS, SI, AGI-III,IV, PICS, Serum lactate, INR≥1.4 and Arterial base excess (ABE≤-6) were significantly correlated with death within 28 days in elderly polytrauma patients with AGI. A multivariable logistic regression of these variables showed that an increasing ISS (OR=2.457,95% CI: 1.962-9.751), AGI-III,IV (OR=4.371,95% CI: 1.857-11.354), and a decreasing GCS (OR=0.436, 95%CI: 0.215-0.574) were each associated with an increasing 28-day mortality in elderly polytrauma patients with AGI (Table 5).

**Figure 2:**
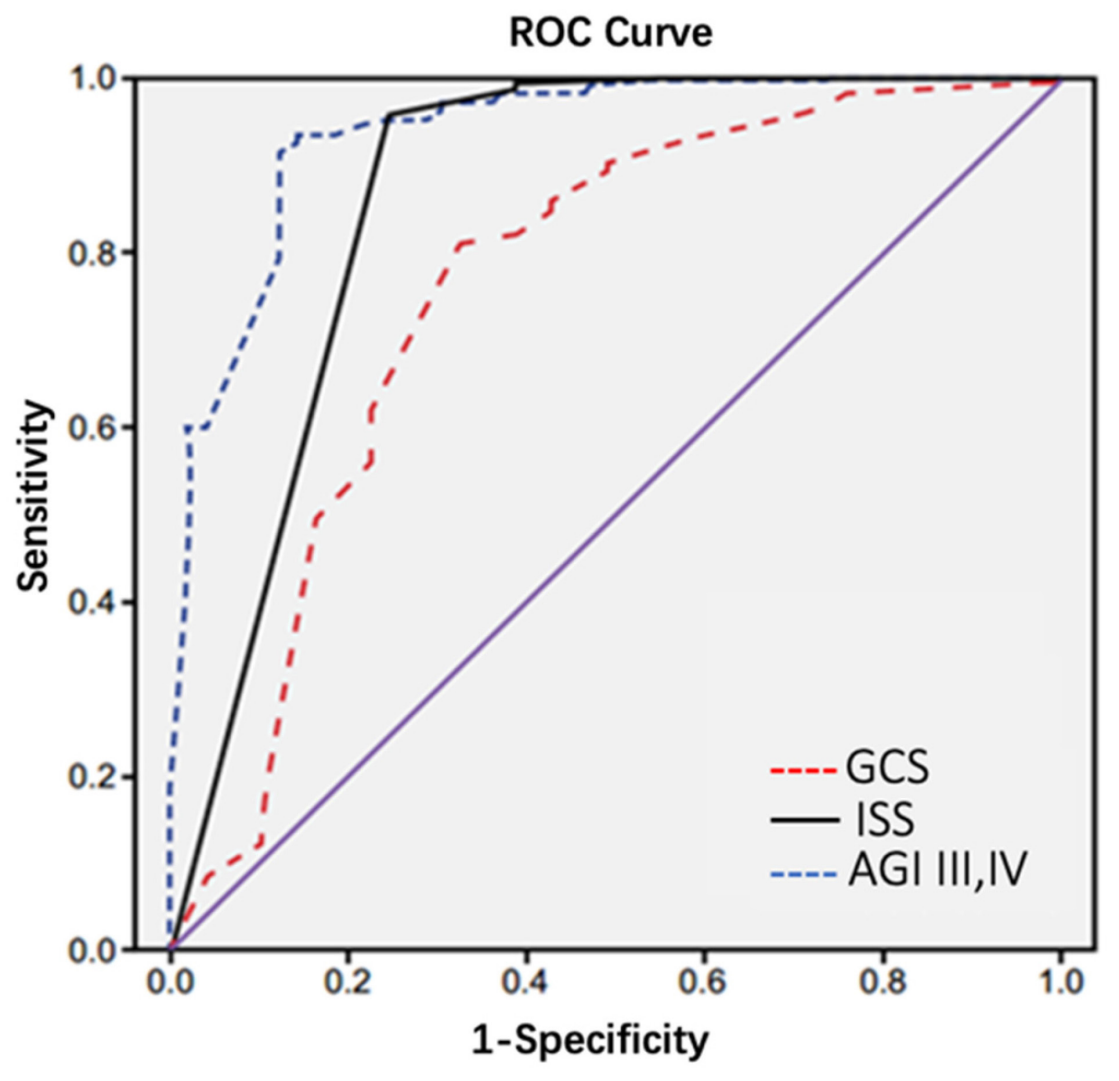
ROC curve of ISS, GCS and AGI-III,IV in 28-day mortality prediction of elderly trauma patients with AGI.

The area under the curve for predicting 28-day mortality in elderly polytrauma patients with AGI was 0.74 for GCS with sensitivity of 80% and specificity of 72%, while for AGI-III,IV was 0.93 with 96% sensitivity and 87% specificity, and for ISS was 0.86 with 97% sensitivity and 80% specificity. AGI-III,IV (93%) had the highest area under ROC for prediction of 28-day mortality. Area under ROC curve for all 3 scores was statistically significant(p<0.001) (Table 6 and Figure 2).

## 4. Discussion

Trauma, particularly in elderly patients, tends to increase with age and is a significant contributor to mortality and disability. This is often due to the decline in physiological function and preexisting comorbidities^13^. Previous studies have shown that elderly trauma accounts for 20.2% of overall trauma cases in all ages, among which over 60% of patients suffer with polytrauma^14^. Of note, compared with young people, even low energy injuries can lead to severe polytrauma in elderly patients^15^. Elderly polytrauma patients exhibit notable distinctions from their younger counterparts in terms of physiology, clinical presentation, and potential complications. Recognizing and addressing these variations is crucial in order to prevent delays in diagnostic procedures, reduce morbidity, and minimize mortality rates^16^. The diminished physiological reserves and organ functionality commonly observed in elderly polytrauma patients can obscure the severity of injuries, complicating the process of clinical evaluation and treatment^16^. For instance, the basic diseases (such as hypertension) and medication (such as beta-blockers, anticoagulants and steroids) hide the normal shock response, resulting to miss haemorrhage and/or hypoperfusion, which is demonstrated in increased mortality with greater age^17^. Previous literatures have reported that overall in-hospital mortality of elderly polytrauma was 36.3%, rising significantly with age, 60.8% occurred in patients aged ≥ 85 which 44.9% were within the first 48h after trauma^18^. In addition, by diminishing physiological reserves, such as pulmonary, gastrointestinal and cardiovascular frailty, complications were at a higher risk in elderly polytrauma. A recent study including 380 elderly polytrauma patients showed that most of the patients (57.4%) had one or more complications and 12.4% of overall died due to severe and fatal complications, such as respiratory failure^18^. Unfortunately, few studies have reported gastrointestinal complications in elderly polytrauma patients. The gut has been postulated as a key factor in the pathogenesis of multiple organ dysfunction syndrome (MODS), with the significance of gastrointestinal failure in MODS having been acknowledged for an extended period of time. Critical illness can precipitate a rapid and profound alteration in the composition of the gut microbiota and activation of the mucosal immune response, potentially leading to bacterial translocation and subsequent gut-derived infections, sepsis, and MODS^19^. Several scoring systems have been developed for MODS, but the gastrointestinal (GI) system has not yet been incorporated into any widely utilized scores.

Recent literatures reported that half of critically ill patients were diagnosed with AGI in the first week of their ICU stay and the patients with AGI have a higher in-hospital mortality (31.1% vs. 18.8%) than those without AGI^20^. Research has shown that elderly polytrauma patients are more susceptible to bacterial and viral infections of the gastrointestinal tract compared to younger individuals, attributed to age-related declines in gastrointestinal function such as reduced gut mobility, decreased bowel absorption, and weakened immune function^21^ ^22^. Unfortunately, there is limited clinical data on the occurrence of AGI in elderly polytrauma patients. As expected, our study indicated that elderly polytrauma patients have a higher risk (73.5%) accompanied by AGI. More importantly, elderly polytrauma patients with AGI were presented relatively high 28-day mortality (40.4%) and super high 60-day mortality (61.2%) compared with elderly group without AGI and youth group with AGI. According to previous results, we infer that several changes occur in gut physiology and immunity, including loss of gut motility, increased bowel wall permeability, and apoptosis of intestinal epithelium in elderly polytrauma patients with AGI, which were characterized by the disorder of digestion and absorption, losing gastrointestinal fluid, and destruction of gastrointestinal mucosal barrier and, finally, those patients usually develop further complications. Those complications may attack repeatedly or just exist persistently during the whole hospitalization period 23. Our study indicated that the four most frequent complications in elderly group with AGI were hospital acquired infections (88.2%), multi-organ failure (66.5%), respiratory failure (56.5) and sepsis (41.6%) which were significantly higher than in elderly without AGI and youth with AGI. These findings suggested that advanced age and AGI may have been linked to occurrence of severely adverse events in polytrauma patients, and even death.

AGI is highly harmful, with atypical symptoms and difficult early diagnosis. And, once it happens, the situation deteriorates quickly 24. In our study, the 28-day mortality rate in the elderly group with AGI was found to be 2-fold higher compared to elderly group without AGI, and while was 4-fold higher compared to young group with AGI. Therefore, early identification of AGI in elderly polytrauma patients is crucial. We evaluated some early parameters between the AGI group and N-AGI group in elderly polytrauma patients. Our study discovered that these elderly patients with AGI were significantly more severely ill (higher ISS scores, shock index and lower GCS scores). The AGI groups also stayed longer in ICU and exhibited higher rates of mechanical ventilation and Use of vasoactive drug than did patients in N-AGI groups. Impaired gastrointestinal motility may lead to aspiration, which further increases the risk of ventilator-associated pneumonia (VAP), and leads to longer mechanical ventilation and ICU hospitalization 25. We found those early indicators after injury such as ISS score, GCS score, shock index, serum lactate, IL-6, and APTT could distinguish between the AGI group and N-AGI group in elderly polytrauma patients. The finding is consistent with the clinical experience in daily work. That is, elderly patients who are more severe are more likely to have higher risk for AGI after polytrauma. The elderly polytrauma patients with traumatic brain injury (TBI) were easy to accompany by gastrointestinal function injury. The reasons cover four aspects: first, the unconsciousness and blood in the mouth and nose resulted in long-term obstruction of the respiratory tract in patients with traumatic brain injury(TBI) ,which leaded to the occurrence of ischemic and hypoxic damage of gastrointestinal mucosa; Second ,central nerve injury in TBI resulted in impaired gastrointestinal nerve function, weakened gastrointestinal peristalsis, abdominal distension, vomiting and other gastrointestinal paralysis symptoms; Third, TBI often needed ventilator treatment that were often accompanied by increased abdominal pressure, which could cause gastrointestinal digestive and absorption dysfunction; Finally, TBI significantly altered the gut microbiome by decreasing commensal bacteria and increasing the presence of pathogenic bacteria to promote disease progression^26^-^28^. Severe shock after polytrauma will lead to low perfusion of gastrointestinal organs (manifested as serum lactate accumulation), and gastrointestinal mucosa will suffer from ischemia and hypoxic injury. In addition, severe polytrauma induced strong stress, excessive inflammatory response (manifested as increased IL-6, TNF-α) and abnormal coagulation function (manifested as increased APTT). IL-6 has been proposed as a prevalent inflammatory marker of intestinal barrier dysfunction, while TNF-α is released early in the inflammatory process, disrupting the intestinal mucosal epithelium and promoting apoptosis of intestinal mucosal epithelial cells through mechanisms such as neutrophil aggregation and release of reactive oxygen and proteolytic enzymes^29^ . TNF-α is crucial in the regulation of gastrointestinal diseases, with its expression in intestinal cells closely associated with intestinal barrier impairment^30^. Age-related alterations in mucosal immune function are of significant importance and may be linked to the equilibrium between inflammatory and anti-inflammatory cytokines. Aging appears to favor the overproduction of proinflammatory cytokines, including IL-6 and TNF-a, which could contribute to the onset of frailty and be correlated with higher rates of illness and death among older individuals^21^.

We observed the risk of mortality in elderly polytrauma patients with AGI. Elderly polytrauma patients were at a massive risk of fatality, with which a low GCS score, injury severity, coagulopathy and acidosis were associated, which is in concordance with the published literature^31^. It is reported that overall mortality rate of elderly polytrauma with TBI was 82.4%. Patients with GCS score between 8 and 12 had higher mortality rate (83% vs 39%) than between 13 and 15, that indicates an influence of GCS score on prognosis^17^. The hypoperfusion and inflammatory cascade is activated in polytrauma patients suffered severe shock. Consequently, metabolic acidosis and hypothermia exacerbate coagulopathy through platelet and coagulation factor consumption and dysfunction, collectively known as the 'lethal triad', leading to a poorer prognosis^32^. Unfortunately, this phenomenon is further compounded in elderly polytrauma patients due to compromised physiological reserves, including renal and cardiovascular frailty 33. It is estimated that 30% to 50% of these chronic critical illness (CCI) patients progress to PICS^7^. As for the population ages, PICS is likely to be next challenging horizon in surgical critical care^34^. Little is known about the combination of PICS with outcome of elderly polytrauma patients with AGI. Our data clearly demonstrate that the PICS increases the risk of in-hospital mortality in elderly polytrauma patients with AGI. Persistent inflammation, immunosuppression and catabolic, and deteriorating nutritional status were mutual transformation or mutual causation due to the pathophysiological characteristics of PICS, which is associated with increased mortality^14^.

The present study again highlight that GI problems translate into worse outcomes, irrespective of cause in elderly patients after polytrauma. GCS, ISS and AGI-III,IV were identified as risk factors for in-hospital mortality of elderly polytrauma patients with AGI. Area under ROC curve using GCS, ISS and AGI-III,IV for predicting death was 0.74, 0.86, and 0.93, respectively; all of these scores were statistically significant in terms of mortality prediction. According to receiver operating characteristic curve (ROC) model used in our study, AGI-III,IV was the strongest predictor of mortality in elderly polytrauma patients with AGI. In alignment with the findings of our research, a recent study involving 470 critically ill patients with acute gastrointestinal injury (AGI) similarly demonstrated that the AGI grading system can effectively indicate the severity of illness. Furthermore, the stratification of AGI into two categories (AGI I + II vs. III + IV) was found to have prognostic significance^24^. These findings collectively suggest that AGI serves as a valuable indicator of critical illness and plays a crucial role in predicting the clinical outcomes of elderly patients following polytrauma.

## Conclusions

Our research examined the frequency, risk factors, and consequences of acute gastrointestinal injury (AGI) in elderly individuals following polytrauma, highlighting the elevated incidence of AGI and its significant association with mortality in this population. It is imperative for trauma specialists to prioritize the care of elderly polytrauma patients with AGI in order to reduce complications and mortality rates.

## Supplementary Material

Supplementary table.

## Figures and Tables

**Table 1:** Patient demographics and characteristics

Characteristics	All patients (n=965)	Youth group (n=746)	Elderly group (n=219)	P
Age, mean (SD), y	50.0±7.6	43.6±8.6	75.1±5.8	<0.001^a^
Gender		-	-	0.955
male, n (%)	644 (66.7)	497 (66.6)	147 (67.1)	-
female, n (%)	321 (33.3)	249 (33.4)	72 (32.9)	-
ISS, mean (SD)	30.9±7.3	30.6±7.5	31.7±8.2	0.062
GCS, mean (SD)	10.4±3.6	10.6±3.8	10.1±3.6	0.084
SI, mean (SD)	0.9±0.4	0.9±0.6	0.8±0.9	0.056
Cause of injury		-	-	0.125
Traffic accident, n (%)	682 (70.7)	524 (70.2)	158 (72.1)	-
high-energy fall, n (%)	219 (22.7)	166 (22.3)	53 (24.2)	-
other, n (%)	64 (6.6)	56 (7.5)	8 (3.7)	-
Injury site		-	-	0.523
Head, n (%)	537 (55.6)	407 (54.6)	130 (59.4)	-
Thorax, n (%)	390 (40.4)	306 (41.0)	84 (38.4)	-
Spine, n (%)	319 (33.4)	255 (34.2)	64 (29.2)	-
Pelvis, n (%)	205 (21.2)	164 (22.0)	41 (18.7)	-
Lower- extremity, n (%)	564 (58.4)	424 (56.8)	140 (63.9)	-
Upper-extremity, n (%)	435 (45.1)	348 (46.6)	87 (39.7)	-
Incidence of AGI, n (%)	614 (63.6)	453 (60.7)	161 (73.5)	<0.001 ^a^
AGI grades, n (%)		-	-	<0.001^ a^
I	297 (48.4)	273 (60.3)	24 (14.9)	-
II	192 (31.3)	128 (28.3)	64 (39.8)	-
III	103 (16.8)	46 (10.1)	57 (35.4)	-
IV	22 (3.5)	6 (1.3)	16 (9.9)	-

**ISS:** injury severity score; GCS: Glasgow coma scale; **SI:** shock index; **AGI:** acute gastrointestinal injury. ^a^P<0.01 denote the elderly group vs. the youth group.

**Table 2:** Variables in AGI group and N-AGI group in elderly polytrauma patient.

Variable	N-AGI group (n=58)	AGI group (n=161)	P
Age, mean (SD), y	76.0±5.4	74.6±5.6	0.101
Gender male, n (%)	- 40 (69.0)	- 118(73.3)	0.646 -
female, n (%)	18 (31.0)	43(26.7)	-
BMI, mean (SD), kg/m2	20.8±4.0	21.2±3.4	0.465
ISS, mean (SD)	28.1±5.9	34.4±6.9	<0.001
GCS, mean (SD)	11.3±4.6	9.3±4.7	0.006
SI, mean (SD)	0.8±0.4	1.2±0.6	<0.001
PICS, n (%)	1 (1.7)	39 (24.2)	<0.001
Heart rate, mean (SD), beats/minute	101.0±21.4	121.6± 24.2	<0.001
Glucose, mean (SD), mmol/L	8.9±3.4	8.7±4.0	0.735
hemoglobin, mean (SD), g/L	116.2±22.8	109.4±25.6	0.076
Serum lactate, median (IQR), mmol/L	1.9 (1.7,2.3)	2.6 (1.8,3.2)	<0.001
ABE≤-6, n (%)	4 (6.9)	35 (21.7)	0.002
PCT, mean (SD), ng/mL	1.5±0.5	5.7±2.4	<0.001
CRP, mean (SD), mg/L	81.4±51.3	92.5±56.4	0.190
IL-6, median (IQR), pg/mL	23.3 (19.6,36.2)	87.3 (49.3,129.5)	<0.001
AST, mean (SD), U/L	45.7±34.2	54.6±34.8	0.095
ALT, mean (SD), U/L	54.8±31.2	61.5±32.9	0.179
Albumin, mean (SD), g/L	32.4±5.1	31.7±6.3	0.448
CYC, mean (SD), mg/L	0.9±0.5	1.0±0.7	0.319
Serum creatinine, median (IQR), μmol/L	79.4 (56.2-89.1)	88.1 (82.8-93.8)	0.523
APTT, mean (SD), s	32.9±9.7	52.1±17.2	<0.001
INR≥1.4, n (%)	7 (12.1)	52 (32.3)	0.005
D dimer, median (IQR), ug/mL	11.3 (9.7,15.5)	24.6 (12.6,36.9)	0.032
Use of vasoactive drug, n (%)	14 (24.1)	98 (60.9)	<0.001
Mechanical ventilation, n (%)	17 (29.3)	113 (70.2)	<0.001
Duration of ICU, median (IQR), days	6 (3,11)	15 (7,22)	<0.001

**BMI:** Body mass index; **ISS:** injury severity score; **GCS:** Glasgow coma scale; **SI:** shock index; **PICS:** persistent inflammation-immunosuppression catabolism syndrome.

**Table 3:** Multivariate analyses of early risk factors for AGI in elderly polytrauma patients.

Variable	P	OR	95%CI
ISS	0.007	2.957	1.285-7.714
GCS	0.032	0.325	0.116-0.906
SI	0.003	2.861	1.372-5.823
Serum lactate, mmol/L	0.001	2.547	1.254-5.028
L-6, pg/mL	0.011	1.771	1.145-8.768
APTT, s	0.024	1.462	1.364-4.254

**ISS:** injury severity score; **GCS:** Glasgow coma scale; **SI:** shock index

**Table 4:** Complications and mortality in polytrauma patients.

Characteristics	All patients (n=965)	Elderly group with AGI (n=161)	Elderly group without AGI (n=58)	Youth group with AGI (n=453)	Youth group without AGI (n=293)
Complications		-	-	-	
AKI	102 (10.6)	28 (17.4)	7 (12.1)	48 (10.6)^c^	19 (6.5)
ARDS	275 (28.5)	91 (56.5)	16 (27.6) ^a^	135 (29.8) ^c^	33 (11.3)^b^
HAI	394 (40.8)	142 (88.2)	21 (36.2) ^a^	175 (38.6) ^c^	56 (19.1)^b^
MOF	233 (24.1)	107 (66.5)	19 (32.8) ^a^	74 (16.3) ^c^	33 (11.3)
Sepsis	154 (16.0)	67 (41.6)	7 (12.1) ^a^	49 (10.8) ^c^	31 (10.6)
Death within 28 days	136 (14.1)	65 (40.4)	14 (24.1) ^a^	41 (9.1) ^c^	16 (5.5)
Death within 60 days	231 (23.9)	99 (61.2)	21 (36.2) ^a^	71 (15.7) ^c^	40 (13.7)

Complications were diagnosed during hospitalization; ^a^P<0.001 denote the elderly group without AGI *vs.* the elderly group with AGI ,and ^b^P<0.001 denote the youth group without AGI vs. the youth group with AGI,^c^ P<0.001 denote the youth group with AGI *vs.* the elderly group with AGI.

**Table 5:** Univariate and multivariate analyses of death within 28 days in elderly polytrauma patients with AGI.

Risk factors	Univariate HR (95% CI)	P	Multivariate OR (95% CI)	P
Age	1.081 (1.063-2.124)	0.001		
ISS	1.457 (1.062-3.751)	0.001	2.457 (1.962-9.751)	0.002
GCS	0.357 (0.143-0.875)	0.021	0.436 (0.215-0.574)	0.017
SI	2.371 (1.142-4.758)	0.011	-	-
AGI-III,IV	3.136 (1.122-5.436)	0.015	4.371 (1.857-11.354)	0.012
PICS	2.863 (1.516-5.062)	0.013	-	-
Serum, lactate	1.874 (1.021-3.539)	0.001	-	-
INR≥1.4	1.274 (1.014-4.057)	0.009	-	-
ABE≤-6	2.471 (1.256-6.539)	0.026	-	-

**CI:** confidence interval; **HR:** hazard ratio; **ISS:** injury severity score; **GCS:** Glasgow coma scale; **SI:** shock index; **AGI-III,IV:** the distribution of the AGI grades; **ABE:** Arterial base excess; **PICS:** persistent inflammation-immunosuppression catabolism syndrome.

**Table 6:** Comparison of sensitivity, specificity and area under the curve based on ROC in ISS, GCS and AGI-III,IV.

Index	Area under the curve	Specificity (%)	Sensitivity (%)	P
GCS	0.74	72	80	<0.001
ISS	0.86	80	97	<0.001
AGI-III,IV	0.93	87	96	<0.001

**ISS:** injury severity score; **AGI-III,IV:** the distribution of the AGI grades; **GCS:** Glasgow coma scale.
