# Supplementary material for: Acute Gastrointestinal Injury in Polytrauma: Special Attention to Elderly Patients
Source: Int J Med Sci. 2024 Aug 26;21(12):2315–23. doi: 10.7150/ijms.98997 (PMC11413898; doi:10.7150/ijms.98997)
Supplement: Supplementary file 1 — Supplementary table. [file ijmsv21p2315s1.pdf]

S1.Acute gastrointestinal injury (AGI) and its different grades

Acute GI injury (AGI) is malfunctioning of the GI tract in critically ill patients due to their acute illness. According to severity the following grades of AGI can be distinguished:

Grades	Rationale	Examples
**AGI grade I** (risk of developing GI dysfunction or failure)—the function of the GI tract is partially impaired, expressed as GI symptoms related to a known cause and perceived as transient	Condition is clinically seen as occurrence of GI symptoms after an insult, which expectedly has temporary and self-limiting nature.	Postoperative nausea and/or vomiting during the first days after abdominal surgery, postoperative absence of bowel sounds, diminished bowel motility in the early phase of shock.
**AGI grade II** (gastrointestinal dysfunction)—the GI tract is not able to perform digestion and absorption adequately to satisfy the nutrient and fluid requirements of the body. There are no changes in general condition of the patient related to GI problems.	The condition is characterized by acute occurrence of GI symptoms requiring therapeutic interventions for achievement of nutrient and fluid requirements. This condition occurs without previous GI interventions or is more severe than might be expected in relation to the course of preceding abdominal procedures.	Gastroparesis with high gastric residuals or reflux, paralysis of the lower GI tract, diarrhoea, intra-abdominal hypertension (IAH) grade I (intra-abdominal pressure (IAP) 12–15 mmHg), visible blood in gastric content or stool. Feeding intolerance is present if at least 20 kcal/kg BW/day via enteral route cannot be reached within 72 h of feeding attempt.
**AGI grade III** (gastrointestinal failure)—loss of GI function, where restoration of GI function is not achieved despite interventions and the general condition is not improving.	Clinically seen as sustained intolerance to enteral feeding without improvement after treatment (e.g. erythromycin, postpyloric tube placement), leading to persistence or worsening of MODS.	Despite treatment, feeding intolerance is persisting—high gastric residuals, persisting GI paralysis, occurrence or worsening of bowel dilatation, progression of IAH to grade II (IAP 15–20 mmHg), low abdominal perfusion pressure (APP) (below 60 mmHg). Feeding intolerance is present and possibly associated with persistence or worsening of MODS.
**AGI grade IV** (gastrointestinal failure with severe impact on distant organ function)—AGI has progressed to become directly and	Situation when AGI has led to an acute critical deterioration of the general condition of the patient with distant organ dysfunction(s).	Bowel ischaemia with necrosis, GI bleeding leading to haemorrhagic shock, Ogilvie' s syndrome, abdominal compartment syndrome (ACS) requiring decompression**.**

immediately life-threatening, with worsening of MODS and shock.		
